# Development of a predictive model using the Kihon Checklist for older adults at risk of needing long‐term care based on cohort data of 19 months

**DOI:** 10.1111/ggi.14456

**Published:** 2022-08-17

**Authors:** Kanae Sato, Shinya Ishii, Michiko Moriyama, Junyi Zhang, Kana Kazawa

**Affiliations:** ^1^ Division of Nursing Science Graduate School of Biomedical and Health Sciences, Hiroshima University Hiroshima Japan; ^2^ Department of Medicine for Integrated Approach to Social Inclusion Graduate School of Biomedical and Health Sciences, Hiroshima University Hiroshima Japan; ^3^ Mobilities and Urban Policy Lab, Graduate School of Advanced Science and Engineering Graduate School for International Development and Cooperation, Hiroshima University Hiroshima Japan

**Keywords:** frailty, good health and well‐being, Kihon Checklist, predictive modeling, risk of long‐term care

## Abstract

**Aim:**

This study developed a risk scoring tool and examined its applicability using data from the Kihon Checklist cohort dataset for 19 months to predict the transition from no certification for long‐term care to long‐term care level 3 or above.

**Methods:**

Data were collected from 26 357 functionally independent, community‐dwelling older adults in a Japanese city who answered the Checklist in 2014 and were followed for 19 months. Individuals certified for long‐term care during the follow‐up period were classified into three levels depending on their certification status: low, moderate, and high long‐term care levels. Relationships between the Kihon Checklist domains and high long‐term care levels were examined using the logistic regression model. A score chart predicting incidents of high long‐term care levels was created to facilitate its applicability.

**Results:**

As of 2016, 971 participants were certified for long‐term care (3.7%), of which 168 (0.6%), 357 (1.4%), and 446 (1.7%) were certified as high, moderate, and low long‐term care levels, respectively. Variables associated with the certification of high long‐term care level included difficulties in activities of daily living, a decline in locomotor and cognitive function in the Kihon Checklist domains, and age. The score chart was created based on these variables and demonstrated excellent discriminatory ability, with an area under curve of 0.817 (95% confidence interval: 0.785–0.849).

**Conclusions:**

The Kihon Checklist can predict the future development of a high degree of dependency. The score chart we developed can be easily implemented to identify older adults at high risk with reasonable accuracy. **Geriatr Gerontol Int 2022; 22: 797–802**.

## Graphical abstract

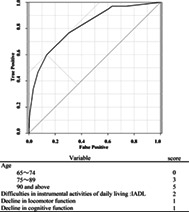

## Introduction

Japan has the highest propotion of older adults (aged 65 years and older) in the world. In 2018, the percentage of older adults was 28.1%, and this value is projected to increase to 38.4% by 2065.^1^ In a super‐aged society, prolonging healthy life expectancy to avoid the need for long‐term care (LTC) is essential for maintaining the social security system.^2^ In 2000, the Japanese government introduced the Long‐term Care Insurance (LTCI) system to support older adults who need LTC.

LTCI data can be used to identify frail individuals at high risk of needing LTC^3^, ^4^, ^5^ in the near future and to provide preventive care and interventions. Approximately 7–10% of community‐dwelling older adults in Japan are frail.^6^, ^7^ Therefore, early identification and intervention for frail older adults are critical for maintaining and improving their quality of life.

Various evaluation tools have been developed to identify frailty in older populations. The Cardiovascular Health Study criteria^8^ and the Frailty Index,^9^ which evaluate physical frailty, have been used internationally. In Japan, the Kihon Checklist (KCL), a 25‐item self‐administered questionnaire, has been widely used to identify older adults at high risk of needing preventive care by screening for multifunctional aspects of frailty, including physical, psychological, social and cognitive frailty.^10^, ^11^, ^12^ Local governments used the KCL to assess all older adults' risk of LTC certification between 2006 and 2015. Since 2016, this has become non‐compulsory, but many local governments have used it continuously in various ways, targeting in particular frail, high‐risk individuals who need intervention.^10^ However, how to use the KCL results' depends on the local government's discretion.

Previous studies using the KCL examined the relationship between the KCL domains and the assessment of frailty using the Comprehensive Geriatric Assessment,^13^ and verified the risk of LTC certification by setting the presence or absence of LTC certification as a dependent variable and the domains of the KCL as independent variables.^12^, ^14^ However, there is no study on the combination of KCL domains. While many studies have focused on the presence or absence of LTC certification, the differences in LTCI care levels have not received much attention.^15^

Tsuji *et al*.^16^ developed a 12‐item risk assessment scale to predict incident functional disability, including all LTC levels among older adults. This scale identifies all levels of LTC certification with good sensitivity and specificity. However, we believe that targeting LTC level 3 or above, described as “*almost all care is required due to a significant decrease in activities of daily living and instrumental activities of daily living (IADL)*”,^17^ is extremely important. This population requires extensive professional care and a considerable increase in per capita expenditure compared with the population with LTC level 2 and below.^18^ Moreover, the Japanese government sets LTC level 3 and above as a criterion for admission to LTC facilities.^19^ The care burden of family caregivers dramatically increases when LTC reaches level 3 or above, which leads to a deterioration in the caregiver's quality of life and health.^20^ In 2018, 21.8% of newly certified older adults with LTC were of level 3 or above.^21^ Because the population is aging so rapidly that there are insufficient numbers of care professionals, it is important to efficiently identify people at risk of shifting to LTC level 3 and to connect them to preventive services at an early stage.

Historically, the use of the KCL in local governments has been to identify older adults in each domain and implement specific preventive programs. However, because multiple factors are involved in the deterioration of their condition, it is necessary to make a comprehensive evaluation by combining KCL domains. Furthermore, previous studies have suggested that there may be differences between risk factors predicting severe (LTC level 3 and above) and mild LTC levels because individual trajectories of deterioration are different in LTC levels among older adults.^22^, ^23^ For populations with different trajectories, interventions tailored to their specific characteristics could be effective. It is most important to develop methods to evaluate older adults comprehensively using a combination of KCL domains associated with severe LTC levels.

Thus, to identify older adults at risk of LTC level 3 or above, we created a predictive model that interprets the results of the KCL by combining each domain and examining the possibility of applying it in clinical settings. A predictive model based on the KCL may enable authorities to effectively apply scientifically sound selection criteria to assist high‐risk older adults in need of preventive care.

Therefore, this study aimed to develop a risk scoring model using 19 months of KCL cohort data to predict the transition of older adults from no certification for LTC to LTC level 3 or above and then to examine the model's ability to estimate the probability of such status. Before developing this tool, we identified the KCL domains that were more likely to shift with each level of LTC status.

## Methods

### Study design

A retrospective cohort study was conducted.

### Participants and procedure

Participants included older residents residing in Kure City, Hiroshima Prefecture, Japan, aged 65+ years at the time of KCL implementation. As of March 2014, the older adult population in Kure city numbered 72 177, accounting for 33.6% of the city's total population.

In June 2014, the KCL was administered to 43 630 older adults in Kure. By August 2014, 28 958 (66.4%) of them had answered the KCL. Subsequently, those who had already been certified for LTC, had completed the KCL, were deceased, had out‐migrated, or had missing data were excluded (*n* = 1833). After matching the KCL data of those who responded to all 25 items in August 2014 with the LTC certificate data in March 2016 and excluding deaths and out‐migration, 26 357 valid responses were analyzed.

### Ethical considerations

As the long‐term care insurer, Kure City provided us with the data, and the analysis was conducted on an opt‐out basis. Opt‐out information was posted from the Hiroshima University website to the public domain. For insured persons, in conformance with the City Personal Protection Regulations, this study was performed as a joint research project with Hiroshima University and formed part of the city's healthcare policies. The ethics committee of Hiroshima University approved this study on December 14, 2016 (No. 578) and October 25, 2021 (No. E‐2644).

### Measures

#### KCL domains

KCL is a self‐administered questionnaire consisting of seven domains (difficulties in IADL, decreased locomotor function, undernutrition, decreased oral function, being homebound, decreased cognitive function, and depressive mood), with 25 questions with yes or no answers. In each domain, there are cut‐off points that are useful for predicting LTC certification.^12^, ^13^

#### LTC: Level classification

The original seven LTC levels (support needs: levels 1 and 2, and care needs: levels 1 to 5)^17^ were reclassified into three levels: (1) low LTC level (LTC‐Low [support needs level 1 or 2: care is not needed but is necessary for maintaining/improving mental and physical functions in the future]); (2) moderate LTC level (LTC‐Moderate [care needs level 1 or 2: care is needed but independent living is possible]); and (3) high LTC level (LTC‐High [care needs level 3 or above: care is needed and independent living is difficult]).

### Statistical analysis

#### Relationship between KCL results (2014) and LTC certification (2016)

First, the number of participants eligible for each KCL domain was calculated with respect to the 26 357 participants who completed the KCL in 2014 and were followed up for 19 months until March 2016. We specified the LTC levels of the participants in March 2016 using the LTC data provided by Kure City. We conducted a chi‐square test to calculate the number and percentage of hits in one or more domains and to determine the association with each LTC certification.

#### Developing a risk scoring tool to predict transition to LTC level 3 and above

First, in a preliminary analysis to assess the additive contribution of the KCL to improve the prediction of transition to LTC‐High, logistic regression models with and without the KCL to estimate the probability of LTC‐High were built and compared. All models were adjusted for age and sex, and the KCL was entered as a total score or the scores calculated in each domain. The additive contribution of the KCL was evaluated using continuous net reclassification improvement (NRI) and integrated discrimination improvement (IDI). NRI quantifies the correct movement in categories, whereas IDI assesses the improvement for integrated sensitivity and specificity.^24^

Second, a multivariate logistic regression model including age, sex, and all KCL domains was constructed. It is common practice among local governments to apply specific cut‐off points to each domain of KCL to determine eligibility for intervention services programs. As previous studies have reported that it is possible to predict new LTC certification using cut‐off points,^11^, ^12^ we decided to adopt cut‐off points and dichotomized all KCL domains.

Backward elimination was implemented, and a shrinkage method was applied to correct the regression coefficients for over‐optimism. Lastly, to enhance its clinical applicability, the final model was presented as a score chart, which was created based on rounded values of the shrunken regression coefficients.

We used the receiver operating characteristic (ROC) curve and calculated the area under the curve (AUC) to evaluate the discriminative ability of each model. The difference in AUC in each model was compared using 1000 bootstrap replications. Sensitivity and specificity were calculated using Youden's index method. We have provided additional multivariate logistic regression analysis results with LTC level 1 and above as the dependent variable and ordinal logistic regression analysis with LTC level 1 to 2 and LTC level 3 and above as dependent variables in Supporting Information Tables S1 and S2. Statistical analyses were performed using IBM SPSS Statistics (ver. 26) and R ver. 4.0.3 (R Core Team [2020]);^25^ *P* < 0.05 was considered to be statistically significant.

## Results

The average age of the participants (*N* = 26 357) was 76.8 (SD ± 6.5; age range: 66–104) years. Dividing age groups into five age categories from 65 and above, people aged 70–74 made up the largest age group (*N* = 7447; 28.3%). In all age groups, there were more women than men (53.0–69.8%).

### Participants' KCL result in 2014 and LTC level in 2016

The numbers and proportions of the study population with different hits for KCL domains in 2014 and those who were certified or not certified for LTC in 2016 are shown in Table 1. Among the 971 participants certified for LTC in 2016 (3.7%), 168 were categorized as LTC‐High (0.6%), 357 as LTC‐Moderate (1.4%), and 446 as LTC‐Low (1.7%). Paticipants who hit for even one KCL domain (hitting even one applicable domain) were more likely to transition to LTC certification.

**Table 1:** Participants' KCL result in 2014 and long‐term care level in 2016

KCL domain	KCL results in 2014 n (%)		Certified LTC benefits in 2016								Not certified in 2016 n (%)	
			Total		LTC‐ High level		LTC‐ Moderate level		LTC‐ Low level			
Total number	26 357	(100.0)	971	(3.7)	168	(0.6)	357	(1.4)	446	(1.7)	25 386	(96.3)
Number of respondents with no impairment	11 255	(100.0)	152	(1.4)	35	(0.3)	59	(0.5)	58	(0.5)	11103	(98.6)
Number of respondents with at least 1 impairment	15 102	(100.0)	819	(5.4)	133	(0.9)	298	(2.0)	388	(2.6)	14283	(94.6)
Difficulties in IADL (range: 1–5, cut‐off ≥3)	2587	(100.0)	339	(13.1)	79	(3.1)	138	(5.3)	122	(4.7)	2248	(86.9)
Decline in locomotor function (range: 1–5, cut‐off ≥3)	4875	(100.0)	508	(10.4)	85	(1.7)	169	(3.5)	254	(5.2)	4367	(89.6)
Undernutrition (range: 1–2, cut‐off ≥2)	401	(100.0)	34	(8.5)	5	(1.2)	17	(4.2)	12	(3.0)	367	(91.5)
Decline in oral function (range: 1–3, cut‐off ≥2)	4332	(100.0)	285	(6.6)	59	(1.4)	90	(2.1)	136	(3.1)	4047	(93.4)
Being homebound (range: 1–2, applicable at not going out more than once a week)	2243	(100.0)	255	(11.4)	52	(2.3)	98	(4.4)	105	(4.7)	1988	(88.6)
Decline in cognitive function (range: 1–3, cut‐off ≥2)	8679	(100.0)	520	(6.0)	101	(1.2)	214	(2.5)	205	(2.4)	8159	(94.0)
Depressive mood (range: 1–5, cut‐off ≥2)	7905	(100.0)	546	(6.9)	85	(1.1)	195	(2.5)	266	(3.4)	7359	(93.1)
Total KCL frailty (range: 1–25, cut‐off ≥7)	7270	(100.0)	653	(9.0)	118	(1.6)	236	(3.2)	299	(4.1)	6617	(91.0)

*Chi‐square test. P* < 0.001. Long‐term care levels were re‐grouped into three: LTC‐Low (support needs level 1 or 2 under Long‐term Care Insurance), LTC‐Moderate (care needs level 1 or 2), and LTC‐High (care needs level 3 or above).

*Note*: Percentage of people in each domain who had a hit in at least one impairment.

IADL, instrumental activities of daily living; KCL, Kihon Checklist, LTC, long‐term care.

Only 152 (1.4%) participants who did not hit any KCL domain in 2014 became LTC‐certified. However, 819 (5.4%) of those who had hits for any domain in 2014 became LTC‐certified, showing a more significant proportion among this group (*P* < 0.001). Regarding all three LTC levels, the number of participants with at least one impairment was significantly higher than the number with no impairment (*P* < 0.001).

Based on LTC levels in 2016, IADL (LTC‐High: 3.1%; LTC‐Moderate: 5.3%) and being homebound (LTC‐High: 2.3%; LTC‐Moderate: 4.4%) were the most common KCL domains for which LTC‐High and LTC‐Moderate participants showed hit responses. However, a decline in locomotor function (5.2%), IADL (4.7%), and being homebound (4.7%) were the most common KCL domains for which participants allocated to the LTC‐Low category showed hit responses.

### Additive contribution of the KCL for the prediction of LTC‐High

Table 2 shows the predictive performance of models with and without the KCL. The addition of the KCL, either as a total score (model 2) or as the score in each domain (model 3), to the model including age and sex (model 1) significantly improved predictive performance. In addition, the model with KCL domain scores (model 3) performed significantly better than that with the KCL total score (model 2) in terms of IDI (*P* < 0.001). IDI showed that model 3 improved average sensitivity and average ‘one minus specificity’ compared with other models.^24^

**Table 2:** Comparison of models using age and sex with models adding KCL in all domains

	AUC	(95% CI)	Sensitivity Specificity	*P*‐value of test for differences in AUC	Continuous NRI (95% CI)	IDI (95% CI)
Age‐ and sex‐adjusted model (Model 1)	0.800	(0.766–0.834)	75.0%	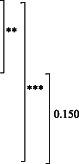	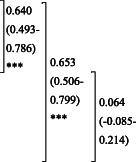	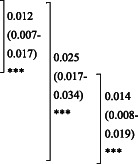
			70.1%			
Model 1 + KCL total score (Model 2)	0.837	(0.806–0.868)	72.0%			
			80.7%			
Model 1 + KCL domain score (continuous variable) (Model 3)	0.846	(0.816–0.877)	73.8%			
			81.2%			

* P < 0.05, ** P < 0.01, *** P < 0. 001. Sensitivity and specificity were calculated using Youden's index method. Bonferroni‐adjusted *P*‐values are shown in the table (the observed *P*‐value was multiplied by the number of comparisons made to adjust for pairwise comparisons).

AUC, area under the curve; IDI, integrated discrimination improvement; KCL, Kihon Checklist; NRI, net reclassification improvement; 95% CI, 95% confidence interval.

### Prediction accuracy of score chart using the selected KCL domains

With LTC‐High as the dependent variable, the results of logistic regression analysis using the backward stepwise selection method showed that age (OR = 1.14; 95% confidence interval (CI): 1.12–1.17), IADL (OR = 3.63; 95% CI: 2.56–5.16), decreased locomotor function (OR = 1.49; 95% CI: 1.06–2.10), and decreased cognitive function (OR = 1.62; 95% CI: 1.16–2.26) were the predictive variables (Table 3). The calculated AUC was 0.839 (95% CI: 0.808–0.869). Sensitivity was 79.8% and specificity was 73.4% when the sum of sensitivity and specificity was maximized.

**Table 3:** Related factors for participants certified for LTC level 3 or above

Variable	Odds ratio	(95% CI)	*P*‐value
Age	1.14	(1.12–1.17)	<0.001***
Difficulties in IADL	3.63	(2.56–5.16)	<0.001***
Decline in locomotor function	1.49	(1.06–2.10)	0.024*
Decline in cognitive function	1.62	(1.16–2.26)	0.005**

* *P* < 0.05, ** P < 0.01, *** P < 0.001. Cut‐off points for the seven domains of KCL: difficulties in IADL (≥3 out of 5 questions); decline in locomotor function (≥3 of 5 questions); decline in cognitive function (≥2 of 3 questions). Multivariate logistic regression analysis using backward stepwise selection method; Hosmer–Lemeshow test *P* = 0.76; AUC was 0.839 (95% CI: 0.828–0.869), sensitivity was 79.8%, and specificity was 73.4%.

IADL, instrumental activities of daily living; 95% CI, 95% confidence interval.

**Figure 1:**
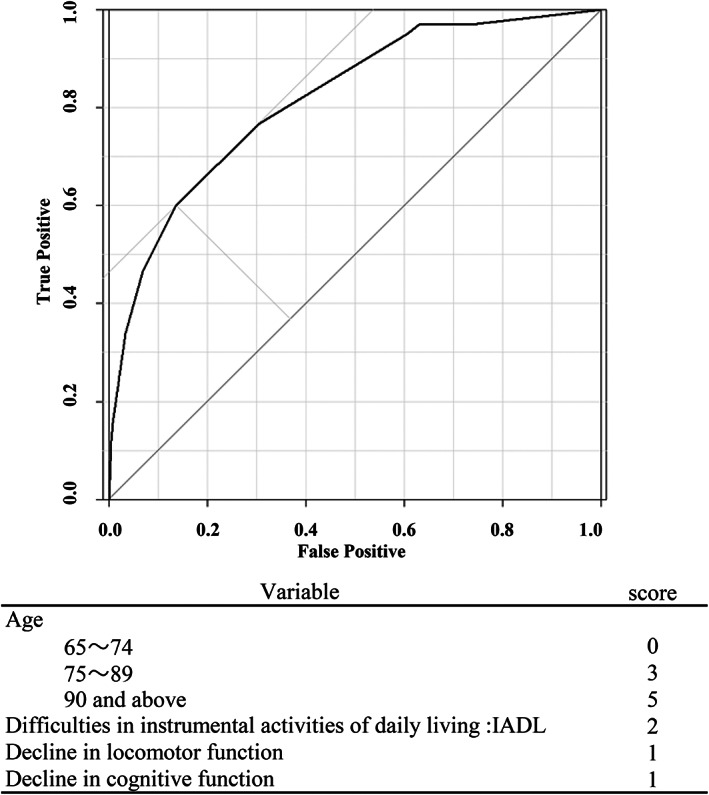
Receiver operating characteristic (ROC) curve for the risk evaluation model. In each domain of the Kihon Checklist (KCL), cases are scored above a cut‐off point: difficulties in activities related to daily life, IADL (≥3 out of 5 questions); decline in locomotor function (≥3 out of 5 questions); decline in cognitive function (≥2 out of 3 questions). Area under the curve (AUC) ROC 0.817 [95% CI 0.785, 0.849]. At a cut‐off point of 5, sensitivity was 60.1% and specificity was 86.2%. At a cut‐off point of 4, sensitivity was 76.8% and specificity was 69.4%. IADL, instrumental activities of daily living; 95% CI, 95% confidence interval.

These four variables were used to create a score chart (Table 4). Age was converted from a continuous to a categorical variable and divided into three groups to make the score chart easy to implement. The score for each variable was calculated based on their odds ratios (Figure 1). Using Youden's index method, the optimal cut‐off values were indicated as 4 points. This score chart can be used to identify at‐risk populations in clinical settings, minimizing the risk of missing them. At a cut‐off point of 4, the sensitivity was 76.8% and specificity was 69.4%. The AUC of the score chart was 0.817, which is lower than that of the model with four selected variables, but is still considered excellent.^26^

**Table 4:** The predictive performance of the score chart using the selected KCL domains

	AUC	(95% CI)	Sensitivity	Specificity
Score chart using Age^†^ and the selected KCL domains (difficulty in IADL, decline in locomotor function, and decline in cognitive function)^‡^	0.817	(0.785–0.849)	60.1%	86.2%

Sensitivity and specificity were calculated using Youden's index method.

^†^ Age was converted to a three‐level categorical variable (65 to 74 years old, 75 to 89 years old, 90 years old and over).

^‡^ Variables were selected after backward elimination.

AUC, area under the curve; IADL, instrumental activities of daily living; KCL, Kihon Checklist; 95% CI, 95% confidence interval.

## Discussion

This study showed that vulnerable older adults who hit any of the KCL domains were at risk of LTC certification. The multivariate logistic regression analysis revealed that IADL, decreased cognitive and locomotor function, and age were independently associated with certification for LTC‐High. Of these, decreased locomotor and cognitive function^14^, ^15^ have been associated with LTC certification in previous studies. Our findings also showed that the IADL domain has the highest OR, and can predict future certification for LTC. Decreased IADL has been associated with the onset of mild cognitive impairment (MCI)^27^ and increased admission to institutions.^28^ Therefore, we understand that IADL is the key to the early detection of the risk of decreased cognitive functioning. Furthermore, we found that the risk of requiring LTC increased with age, which is strongly related to LTC certification, as pointed out in a previous study.^4^

The KCL has been used primarily to select a wide range of frail older adults for community‐based preventive programs by the municipal government. The key to implementing the results of this study in municipal governments is the ability to determine the risk of the target population and prioritize interventions. Considering the trade‐off between this clinical feasibility and the predictive accuracy of models, we believe that the score chart is easy to use for municipal governments.^29^ The novel score chart predicting the risk of LTC level 3 or above in the future has higher specificity than other models and may contribute to efficient targeting and cost‐effective interventions for large populations. In addition, risk scoring based on multi‐domain assessments supports decision‐making related to prioritizing and combining preventive interventions for individuals. For example, for people younger than 75 years, if the person hits the three domains of KCL, namely IADL, decreased locomotor function, and decreased cognitive function, then the total would be 5 points, which exceeds the cut‐off of 4; hence, such individuals can be judged to be at risk of LTC level 3 or above. Similarly, for people aged 75–89 years, having a hit in one of the three domains of the KCL can be considered high risk. Utilizing this model makes it possible to correctly identify and intervene among older adults in need of preventive measures for LTC. Our study contributes to advanced research on future efforts related to LTC prevention, public health, and medical policy‐making.

There are several limitations to this study. First, the analysis was based on data from only 57% of insured persons of Kure city's older adults, owing to missing or untraceable data. Research from a different perspective is needed to determine why older adults did not respond to the KCL. A better understanding of the reasons for older adults requiring LTC, such as illness or family situation, may enable high‐risk individuals to be targeted and for early intervention to be provided. Second, this study used data from older adults living in one area. As risk characteristics are expected to vary between regions, it is necessary to replicate the study using data from different regions.

We propose to identify older adults at high risk of requiring increased levels of LTC and intervention in the future. Therefore, it is worth conducting intervention studies to prevent LTC n among high‐risk populations in selected communities identified by our modeling results.

## Disclosure statement

The authors declare no conflicts of interest.

## Supporting information


**Table S1**. Multivariate logistic regression analysis with LTC level 1 and above as the dependent variable

**Table S2**. Ordinal logistic regression analysis with LTC levels 1 to 2 and LTC level 3 and above as dependent variables

## Data Availability

This study was conducted as the insurer's project (Kure City) in accrdance with the personal information protection ordinance. Therefore, the dataset analyzed in this study are not publicly available.
