# Supplementary material for: Development of a predictive model using the Kihon Checklist for older adults at risk of needing long‐term care based on cohort data of 19 months
Source: Geriatr Gerontol Int. 2022 Aug 17;22(9):797–802. doi: 10.1111/ggi.14456 (PMC9546004; doi:10.1111/ggi.14456)
Supplement: Supplementary file 1 — Table S1. Multivariate logistic regression analysis with LTC level 1 and above as the dependent variable [file GGI-22-797-s002.docx]

Table SuppInfo 1. Multivariate logistic regression analysis with LTC level 1 and above as the dependent variable

| **Variable** | **Odds ratio** | **(95%** **CI)** | ***p*-value** |
| --- | --- | --- | --- |
| Age | 1.14 | (1.12-1.16) | <0.001^***^ |
| Difficulties in IADL | 2.77 | (2.22-3.44) | < 0.001^***^ |
| Decline in locomotor function | 1.47 | (1.20-1.79) | < 0.001^***^ |
| Being homebound | 1.46 | (116-1.84) | 0.001^**^ |
| Decline in cognitive function | 1.80 | (1.48-2.18) | < 0.001^***^ |

*IADL*: Activities related to daily life, *95% CI*: 95% confidence interval

Cut-off points for the seven domains of KCL: difficulties in IADL (≥ 3 out of 5 questions); decline in locomotor function (≥ 3 of 5 questions); being homebound (applicable at not going out more than once a week); decline in cognitive function (≥ 2 of 3 questions)

Using the backward stepwise selection method; Brier score was 0.018; AUC was 0.837 (95% CI: 0.820-0.854), sensitivity was 81.9% and specificity was 71.2%.

*^**^* *p* < 0.01, *^***^* *p* < 0.001
