# Supplementary material for: Development of a predictive model using the Kihon Checklist for older adults at risk of needing long‐term care based on cohort data of 19 months
Source: Geriatr Gerontol Int. 2022 Aug 17;22(9):797–802. doi: 10.1111/ggi.14456 (PMC9546004; doi:10.1111/ggi.14456)
Supplement: Supplementary file 2 — Table S2. Ordinal logistic regression analysis with LTC levels 1 to 2 and LTC level 3 and above as dependent variables [file GGI-22-797-s001.docx]

Table SuppInfo 2. Ordinal logistic regression analysis with LTC　level 1 to 2 and LTC　level 3 and above as dependent variables

| **Variable** | **Odds ratio** | **(95%** **CI)** | ***p*-value** |
| --- | --- | --- | --- |
| Age | 1.13 | (1.12-1.16) | 0.001^**^ |
| Difficulties in IADL | 2.79 | (2.24-3.46) | < 0.001^***^ |
| Decline in locomotor function | 1.47 | (1.20-1.79) | < 0.001^***^ |
| Being homebound | 1.46 | (116-1.84) | < 0.001^***^ |
| Decline in cognitive function | 1.80 | (1.48-2.18) | < 0.001^***^ |

*IADL*: Activities related to daily life, *95% CI*: 95% confidence interval

Cut-off points for the seven domains of KCL: difficulties in IADL (≥ 3 out of 5 questions); decline in locomotor function (≥ 3 out of 5 questions); being homebound (applicable at not going out more than once a week); decline in cognitive function (≥ 2 out of 3 questions)

Brant test was conducted to confirm the proportional odds assumption; *p* ≥ 0.05 for all variables. The Lipsitz test a goodness of fit; *p* < 0. 001

*^**^* *p* < 0.01, *^***^* *p* < 0.001
